# Six Innexins Contribute to Electrical Coupling of *C. elegans* Body-Wall Muscle

**DOI:** 10.1371/journal.pone.0076877

**Published:** 2013-10-09

**Authors:** Ping Liu, Bojun Chen, Zeynep F. Altun, Maegan J. Gross, Alan Shan, Benjamin Schuman, David H. Hall, Zhao-Wen Wang

**Affiliations:** 1 Department of Neuroscience, University of Connecticut Health Center, Farmington, Connecticut, United States of America; 2 Department of Neuroscience, Albert Einstein College of Medicine, Bronx, New York, United States of America; 3 Undergraduate Summer Research Internship Program, University of Connecticut Health Center, Farmington, Connecticut, United States of America; National Institute of Biological Sciences, Beijing, China

## Abstract

*C. elegans* body-wall muscle cells are electrically coupled through gap junctions. Previous studies suggest that UNC-9 is an important, but not the only, innexin mediating the electrical coupling. Here we analyzed junctional current (*I*_*j*_) for mutants of additional innexins to identify the remaining innexin(s) important to the coupling. The results suggest that a total of six innexins contribute to the coupling, including UNC-9, INX-1, INX-10, INX-11, INX-16, and INX-18. The *I*_*j*_ deficiency in each mutant was rescued completely by expressing the corresponding wild-type innexin specifically in muscle, suggesting that the innexins function cell-autonomously. Comparisons of *I*_*j*_ between various single, double, and triple mutants suggest that the six innexins probably form two distinct populations of gap junctions with one population consisting of UNC-9 and INX-18 and the other consisting of the remaining four innexins. Consistent with their roles in muscle electrical coupling, five of the six innexins showed punctate localization at muscle intercellular junctions when expressed as GFP- or epitope-tagged proteins, and muscle expression was detected for four of them when assessed by expressing GFP under the control of innexin promoters. The results may serve as a solid foundation for further explorations of structural and functional properties of gap junctions in *C. elegans* body-wall muscle.

## Introduction

Gap junctions are intercellular channels connecting the cytoplasm of adjacent cells. They are formed by connexins in mammals but innexins in invertebrates. Innexins are homologues of mammalian pannexins [1-3], which may form hemichannels [1,4-11]. Deficiencies and mutations in gap junction proteins cause a variety of human disorders such as demyelination [12,13] ,cataracts [14,15], deafness [16,17], and heart malformations [18].

Each species generally has multiple genes encoding gap junction proteins. For example, the human genome has 21 connexins and 3 pannexins. The *Caenorhabditis elegans* and *Drosophila melanogaster* genomes have 25 and 8 innexins, respectively [19,20]. Different gap junction proteins are often expressed in the same cells, and have the potential to coassemble into functional units, which greatly increases the structural and functional diversity of gap junctions. Analyses using transfected mammalian cell lines and the *Xenopus* oocyte heterologous expression system have shown that many gap junction proteins may coassemble to form heterotypic or heteromeric gap junctions [21]. There is also evidence that heterotypic or heteromeric gap junctions exist in native tissues [21-25]. However, molecular identification and functional attributions for all gap junction proteins expressed in individual cell types have been hampered by a scarcity of specific antibodies and a lack of specific pharmacological inhibitors.

*C. elegans* is a particularly facile model system for understanding the structure and function of gap junctions for several major reasons, including their small number of somatic cells (959 cells) per animal, existence of mutants for all innexin genes, ease to assess protein expression and subcellular localization patterns, and availability of electrophysiological, cell biological and behavioral assays for gap junction functions. Studies with *C. elegans* have uncovered previously unknown functions of gap junctions, such as instructing defecation motor steps by propagating Ca^2+^ waves between enteric muscle cells [26], regulating asymmetric gene expression in selected neurons [27], facilitating synchronous pharyngeal muscle contractions [28,29], promoting sperm guidance to the fertilization site [30], synchronizing body-wall muscle action potentials and Ca^2+^ transients [31], and facilitating spicule thrusts by transmitting signals among male-specific sex muscles [32].

Body-wall muscle occupies a unique niche in gap junction research with *C. elegans* because it is the only tissue for which the dual voltage- and current- clamp techniques have been adapted to analyze physiological and biophysical properties of gap junctions. Previous studies have shown that muscle cells are electrically coupled in a highly organized pattern through low-conductance gap junctions, and that the innexin UNC-9 serves as an important component of the muscle gap junctions [31,33,34]. However, other innexin(s) must be contributing to the muscle coupling because *I*_*j*_ between adjoining muscle cells within the same quadrant is decreased but not absent in the *unc-9* null mutant [33]. To fully understand the function and molecular compositions of the muscle gap junctions, it is important to identify all the innexins functioning in the muscle. In the present study, we identified a total of six innexins contributing to electrical coupling of the muscle through electrophysiological analyses of innexin mutants. The identification of so many gap junction proteins functioning in a single type of cells is unprecedented.

## Results

### Six innexins are required for normal electrical coupling of body-wall muscle cells

Results of our previous study suggest that UNC-9 is not the only innexin forming gap junctions in *C. elegans* body-wall muscle [33]. To identify the remaining innexin(s) functioning in the muscle, we analyzed *I*_*j*_ in additional innexin mutants. Although mutants have been isolated for all the 25 innexins of *C. elegans*, homozygous mutants of 3 innexins (*inx-3, inx-12, inx-13*) are unsuitable for electrophysiological analyses because they are either lethal or sterile. We therefore focused on mutants of the remaining 22 innexins, including *inx-1*(*tm3524*)*, inx-2*(*ok376*)*, inx-4/che-7*(*ok2373*)*, inx-5*(*ok1053*)*, inx-6*(*rr5*)*, inx-7*(*tm2378*)*, inx-8*(*gk42*)*, inx-9*(*ok1502*)*, inx-10*(*ok2714*)*, inx-11*(*ok2783*)*, inx-14*(*ag17*)*, inx-15*(*tm3394*)*, inx-16*(*tm1589*)*, inx-17*(*tm3839*)*, inx-18*(*ok2454*)*, inx-19/nsy-5*(*tm1896*)*, inx-20*(*ok426*)*, inx-21*(*ok2524*)*, inx-22*(*tm1661*)*, eat-5*(*ad464*), *unc-7*(*e5*), and *unc-9*(*fc16*). The *unc-9* mutant was also included for the purpose of comparisons. Molecular lesions of these mutants can be found at the Wormbase website (www.wormbase.org. Accessed 2013 July 1).

**Figure 1:**
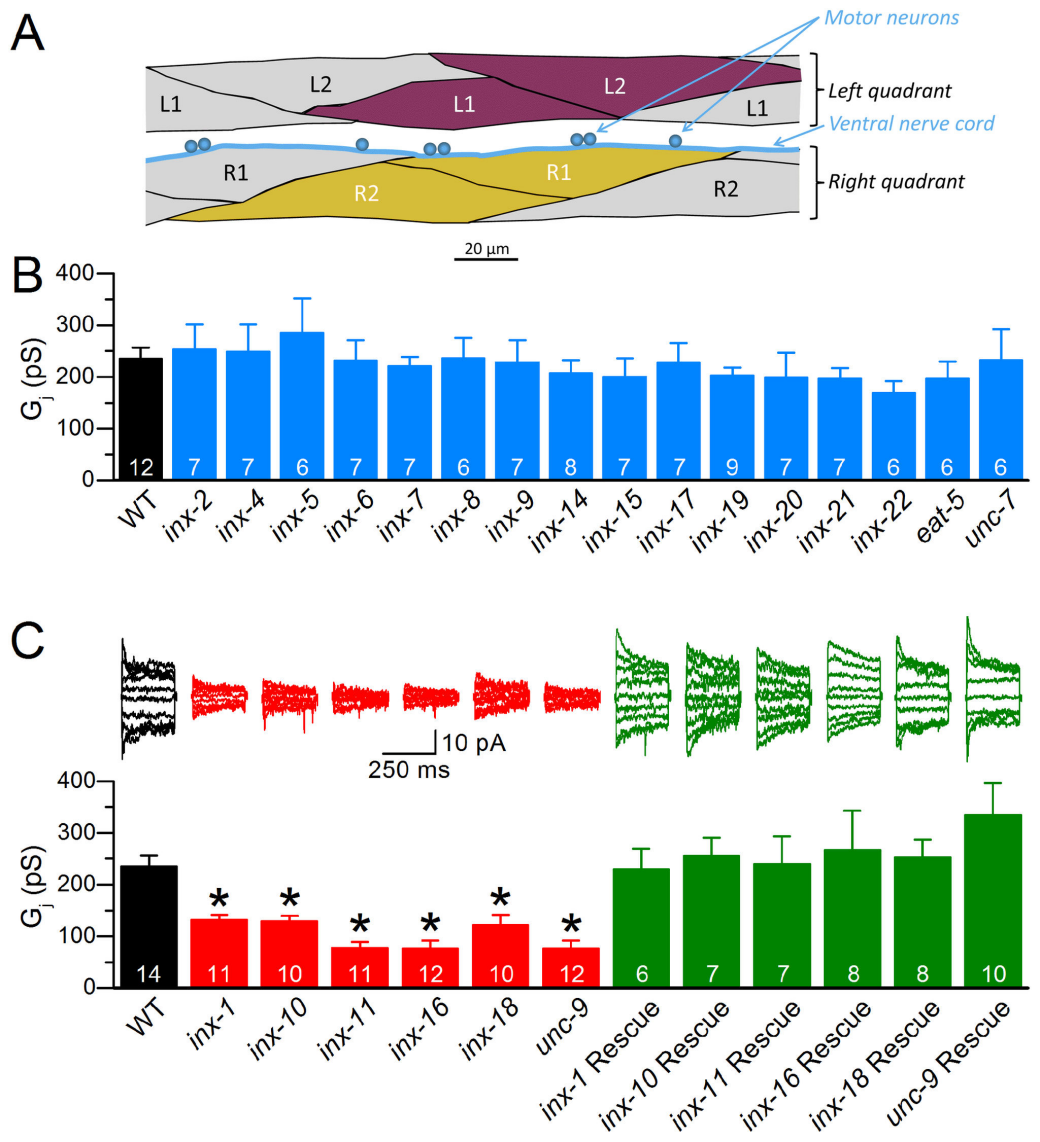
Junctional conductance (*G*_*j*_) of *C. elegans* body-wall muscle was significantly decreased in mutants of six innexins. **A**. Diagram showing ventral body-wall muscle cells. Ventral muscles include the left and right quadrants with each quadrant consisting of two rows of muscle cells in a monolayer. The highlighted cell pairs (L1/L2 and R1/R2) represent those used for electrophysiological analyses. **B**. *G*_*j*_ was indistinguishable between wild type (WT) and mutants of 16 innexins. **C**. Mutants of 6 innexins showed significantly lower *G*_*j*_ when compared with WT, and the coupling defect was rescued completely by expressing a wild-type innexin in each corresponding mutant. The asterisk (*) indicates a statistically significant difference compared with WT. The number on each bar represents the number (*N*) of cell pairs analyzed.

We chose the R1/R2 and L1/L2 pairs of ventral body-wall muscle cells (Figure **1A**) for analyses because their relatively strong electrical coupling made it easier to detect mutant effects. Data from the two cell pairs were combined in subsequent statistical analyses because properties of electrical coupling are indistinguishable between these two cell pairs [33]. Mutants of 16 innexins were similar to wild type in junctional conductance (*G*_*j*_) (Figure **1B**), suggesting that these innexins are not important for electrical coupling. In contrast, mutants of 6 innexins, including *unc-9*, *inx-1, inx-10, inx-11, inx-16* and *inx-18*, displayed a significant decrease in *G*_*j*_ compared with wild type (Figure **1C**), suggesting that these innexins may be required for normal electrical coupling of the muscle. The observations with the *unc-7* and *unc-9* mutants were consistent with those of our previous studies [31,33,34]. Among the six mutants showing deficient muscle electrical coupling, *unc-9*(*fc16*) is due to a premature stop in the intracellular loop between the second and third membrane spanning domains [35] whereas the remaining mutants have either deletion or deletion plus insertion in an innexin gene (www.wormbase.org. Accessed 2013 July 1). We refer to the six mutants as *lf* (loss-of-function) mutants in the remainder of the text.

### Innexins function cell-autonomously to contribute to muscle electrical coupling

The coupling defects described above likely resulted from a deficiency of the innexins in body-wall muscle. Indeed, this has been confirmed for *unc-9*(*lf*) [33,34]. For mutants of the other five innexins, however, the observed muscle coupling defects could also result from other potential causes, such as an indirect effect from non-muscle cells, or mutation of another gene. To determine whether the coupling defect resulted from innexin deficiency in body-wall muscle, we performed muscle-specific rescue experiments for each mutant by expressing the corresponding wild-type innexin under the control of the muscle-specific *myo-3* promoter (P*myo-3*) [36]. In all cases, *G*_*j*_ was restored to wild-type level in the mutant (Figure **1C**), suggesting that all the six innexins function cell-autonomously to contribute to the electrical coupling. Intriguingly, none of the rescued strains showed a significantly higher *G*_*j*_ than wild type, suggesting that overexpression of a single innexin in body-wall muscle is not enough to cause a significant increase in functional gap junctions.

### Innexins may function as two distinct populations of gap junctions

**Figure 2:**
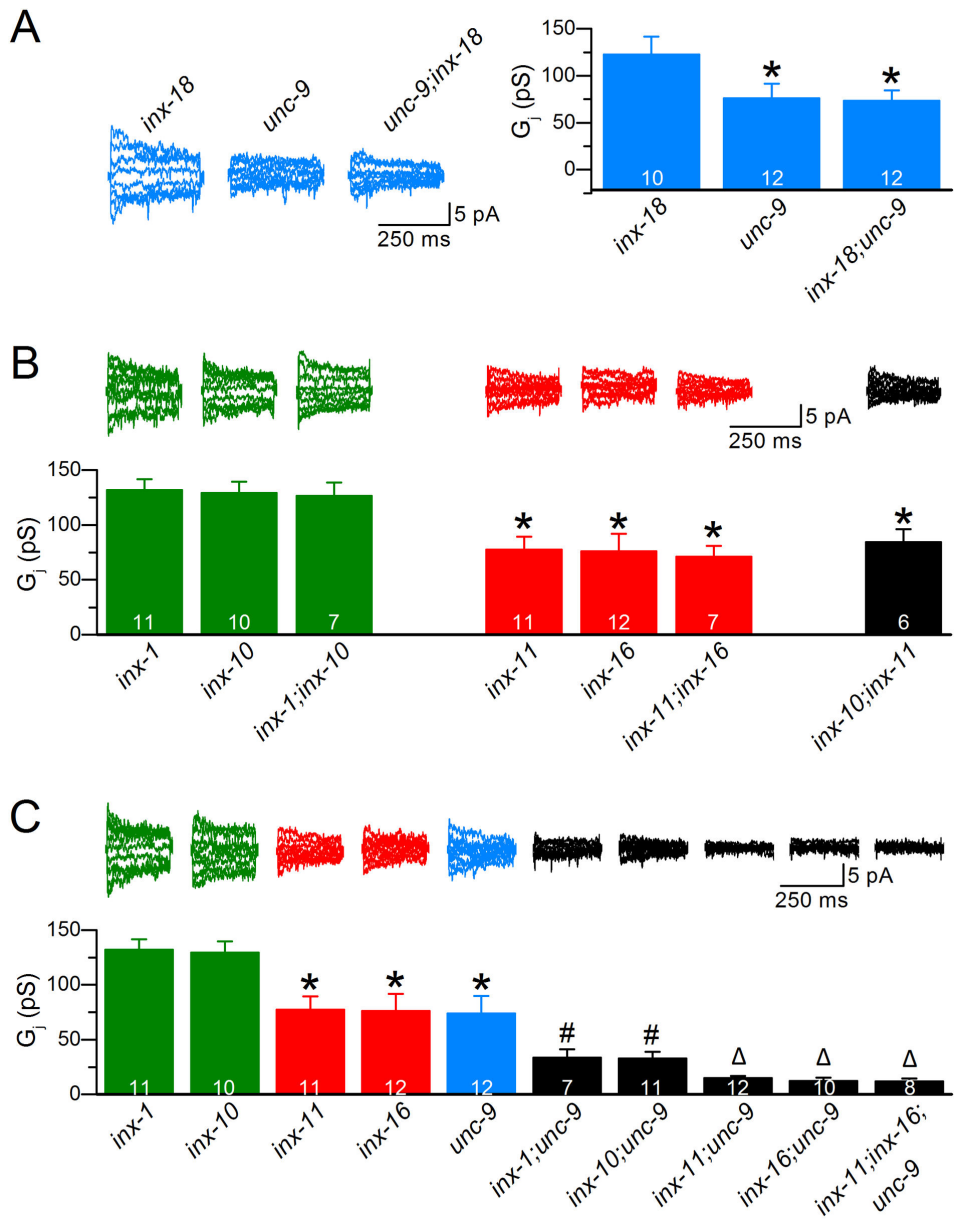
Innexins likely assemble into two distinct populations of gap junctions in *C. elegans* body-wall muscle. **A**. Junctional conductance (*G_j_*) was not further decreased in the *unc-9;inx-18* double mutant compared with the *unc-9* single mutant, suggesting that UNC-9 and INX-18 function together. **B**. *G*_*j*_ was decreased to a similar degree in the *inx-1;inx-10* and *inx-11;inx-16* double mutants compared with their corresponding single mutants although *G*_*j*_ was significantly lower in the mutants of *inx-11* and *inx-16* than those of *inx-1* and *inx-10*. *G*_*j*_ was not further decreased in the *inx-10;inx-11* double mutant, suggesting that the four innexins (INX-1, INX-10, INX-11 and INX-16) likely function together. **C**. Combining selected mutants from A and B resulted in a further decrease of *G*_*j*_, suggesting that there are two distinct populations of gap junctions in muscle. In **A**, the asterisk (*) indicates a statistically significant difference compared with the *inx-18* single mutant. In both B and C, the asterisk indicates a statistically significant difference compared with the single and double mutants of *inx-1* and *inx-10*; the pound sign (#) indicates a statistically significant difference compared with the single and double mutants of *inx-11* and *inx-16*; and the triangle (Δ) indicates a statistically significant difference compared with *inx-1;unc-9* and *inx-10;unc-9*. The number on each bar represents the number (*N*) of cell pairs analyzed.

The six innexins identified through the electrophysiological analyses unlikely form independent populations of homotypic gap junctions because the combined decrease of *G*_*j*_ of these mutants was far greater than 100%. To assess how many populations of gap junctions that these innexins might form in body-wall muscle, we compared *G*_*j*_ among various single and double mutants of the innexins. If two innexins functioned together, *G*_*j*_ in the double mutant would be comparable to that in either single mutant. Otherwise, *G*_*j*_ would be decreased to a larger degree in the double mutant than in either single mutant. We first compared *G*_*j*_ between single and double mutants of *unc-9*(*lf*) and *inx-18*(*lf*) because coupling deficiencies were observed in these two mutants well before we embarked on the project to systematically analyze essentially all innexin mutants. We found that, while *unc-9*(*lf*) caused a larger decrease in *G*_*j*_ than *inx-18*(*lf*), *G*_*j*_ in the double mutant was similar to that in the *unc-9*(*lf*) single mutant (Figure **2A**), suggesting that UNC-9 and INX-18 likely contribute to a single population of gap junctions. The lesser coupling deficiency in *inx-18*(*lf*) than *unc-9*(*lf*) might be because UNC-9 but not INX-18 may form partially functional gap junctions in the absence of its counterpart.

We next compared *G*_*j*_ between single and double mutants for two other pairs of innexins, including INX-1/INX-10 and INX-11/INX-16 because *G*_*j*_ was essentially identical between *inx-1*(*lf*) and *inx-10*(*lf*), and between *inx-11*(*lf*) and *inx-16*(*lf*). We found that *G*_*j*_ was indistinguishable between the *inx-1*(*lf*);*inx-10*(*lf*) double mutant and either *inx-1*(*lf*) or *inx-10*(*lf*) single mutant, and also between the *inx-11*(*lf*);*inx-16*(*lf*) double mutant and either *inx-11*(*lf*) or *inx-16*(*lf*) single mutant (Figure **2B**). These observations suggest that INX-1 and INX-10 likely function together, and so do INX-11 and INX-16. The *G*_*j*_ was significantly lower in the single or double mutants of *inx-11* and *inx-16* than those of *inx-1* and *inx-10* (Figure **2B**). To determine whether there is any functional relationship between these two pairs of innexins, we compared *G*_*j*_ between the double mutant *inx-10*(*lf*);*inx-11*(*lf*) and either single or double mutants of *inx-11*(*lf*) and *inx-16*(*lf*), and found that *G*_*j*_ was not further decreased in the *inx-10*(*lf*);*inx-11*(*lf*) double mutant (Figure **2B**). These analyses collectively suggest that four innexins, including INX-1, INX-10, INX-11 and INX-16, likely contribute to the function of a single population of gap junctions. The lesser coupling deficiency in *inx-1*(*lf*) and *inx-10*(*lf*) than *inx-11*(*lf*) and *inx-16*(*lf*) might be because INX-11 and INX-16 but not INX-1 and INX-10 may form partially functional gap junctions in the absence of their counterparts.

We then determined whether the two populations of gap junctions described above actually belong to a single population by comparing *G*_*j*_ between single, double, and triple mutants of selected innexins, including *unc-9, inx-1, inx-10, inx-11* and *inx-16*. Combining either *inx-11*(*lf*), *inx-16*(*lf*), or both with *unc-9*(*lf*) resulted in a *G*_*j*_ much smaller than that in any of the single mutants (Figure **2C**). Indeed, *I*_*j*_ was essentially indistinguishable from baseline noises in the double and triple mutants. *G*_*j*_ was also further decreased when combining either *inx-1*(*lf*) or *inx-10*(*lf*) with *unc-9*(*lf*) (Figure **2C**). These observations suggest that there are likely two distinct populations of gap junctions in body-wall muscle, with one population consisting of UNC-9 and INX-18 and the other consisting of INX-1, INX-10, INX-11 and INX-16.

### GFP transcriptional fusions show muscle expression of several innexins

**Figure 3:**
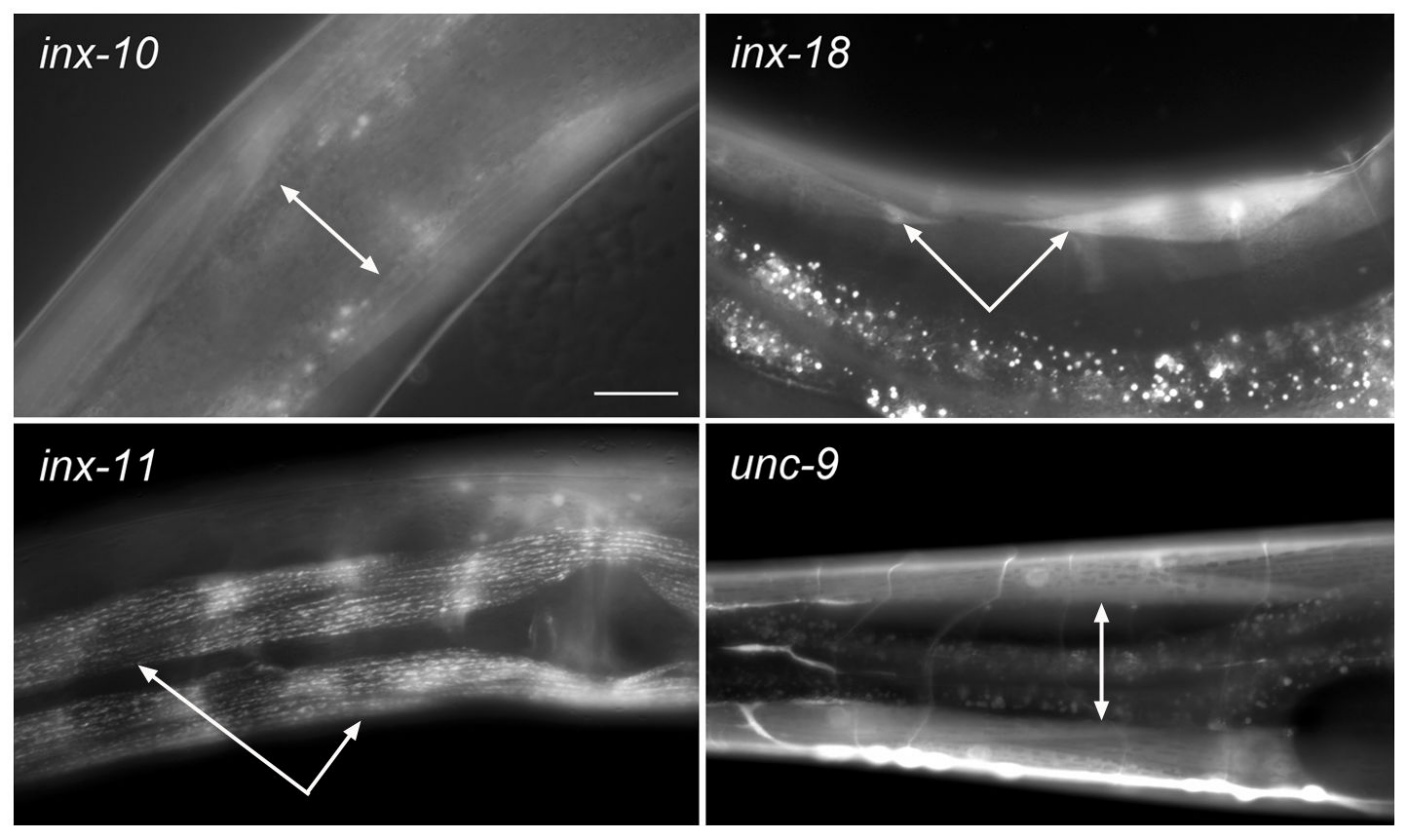
Expression patterns of innexins functioning in *C. elegans* body-wall muscle. Expression patterns were assessed by analyzing GFP signal in live worms expressing innexin promoter and GFP transcriptional fusions. Body-wall muscle expression (indicated by arrows) was observed for four of the six innexins important to muscle electrical coupling, including *unc-9, inx-10, inx-11* and *inx-18*. The *unc-9* expression data were shown previously [33,34].

The electrophysiological data suggest that six innexins are expressed in body-wall muscle. To assess muscle innexin expression using an independent approach, we expressed GFP as innexin promoter::GFP transcriptional fusions and analyzed GFP signal in live worms. We first used a homologous recombination approach for all the 25 innexins to detect potential muscle expression as part of an effort to provide a high resolution map for all the 25 innexins [37]. Specifically, a plasmid containing a short promoter sequence (typically ~1 kb) fused to GFP coding sequence was made for each innexin and coinjected with a cosmid containing the corresponding innexin gene. It was expected that homologous recombination *in vivo* would result in a promoter::GFP transcriptional fusion containing the entire innexin promoter [38,39]. Body-wall muscle expression of several innexins, including *unc-9, inx-8, inx-10, inx-11, inx-14, inx-18*, *inx-19 and inx-21*, was detected using this approach. The muscle expression of *inx-10, inx-11, inx-18* and *unc-9* is shown in Figure **3**. The muscle expression of the other innexins is not shown because those innexins did not appear to play a role in electrical coupling.

The experiments described above failed to confirm body-wall muscle expression for *inx-1* and *inx-16*. One possible cause was that a full-length innexin promoter was not assembled through homologous recombination *in vivo* due to unexpected deletions in the cosmid or other factors. To address this possibility, we made promoter::GFP transcriptional fusions for both innexins by fusing a longer promoter sequence to GFP, and examined GFP expression patterns in transgenic worms. The length of promoter sequence used was 4925 bp for *inx-1* and 1931 bp for *inx-16*. The length of the *inx-16* promoter sequence used was relatively short because the translation initiation site of *inx-16* is very close to the coding region of another upstream gene (~1.0 kb). However, GFP expression was still not observed in body-wall muscle cells (not shown).

### Innexins are localized to intercellular junctions

**Figure 4:**
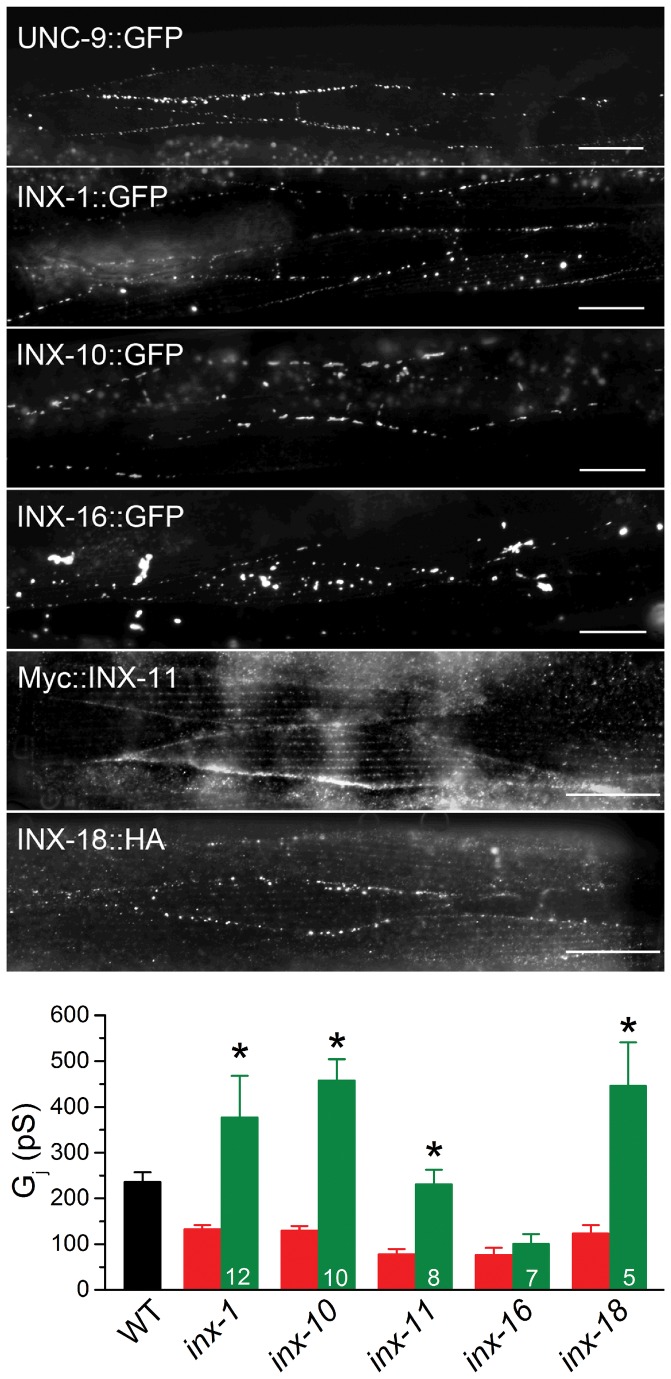
Subcellular localization of innexins in *C. elegans* body-wall muscle. ***Top***, Subcellular localization patterns of innexin fusion proteins in body-wall muscle. INX-1::GFP, INX-10::GFP, Myc::INX-11, and INX-18::HA mainly showed punctate localization at muscle intercellular junctions. INX-16::GFP showed some punctate localization between muscle cells but mainly intracellular aggregates. ***Bottom***, Effects of the fusion proteins (expressed in corresponding mutants) on body-wall muscle electrical coupling. The asterisk (*) indicates a statistically significant difference compared with the corresponding mutant (paired *t*-test). The numbers inside the columns represent sample size (*n*). The wild-type (WT) and mutant data in Figure 1C were included for comparisons.

Gap junctions typically appear as plaques/puncta at the plasma membranes between adjacent cells, which has been shown for UNC-9 by immunostaining using an UNC-9-specific antibody and by expressing GFP-tagged full-length UNC-9 [33,34]. We therefore analyzed subcellular localization for the remaining five innexins identified by the electrophysiological analyses. We first expressed GFP-tagged full-length innexins in body-wall muscle under the control of P*myo-3* and analyzed subcellular localization patterns of the fusion proteins. With respect to INX-1, two different splice isoforms (INX-1a and INX-1c/GenBank KF137642) were analyzed. GFP fluorescent puncta were observed at muscle intercellular junctions with INX-1c::GFP, INX-10::GFP and INX-16::GFP although the size and density of the fluorescent puncta varied among them and INX-16::GFP mostly appeared as intracellular aggregates (Figure **4**, *top*). However, INX-1a::GFP showed diffuse muscle expression; and INX-11::GFP and INX-18::GFP were not localized to intercellular junctions but mainly appeared as large intracellular aggregates (not shown). We reasoned that the large intracellular aggregates might reflect a GFP-induced trafficking defect, and that a smaller epitope tag might have a lesser problem of this sort. Therefore, we expressed Myc-tagged INX-11 (Myc::INX-11) and HA-tagged INX-18 (INX-18::HA) in muscle cells under the control of P*myo-3* and analyzed subcellular localization of the tagged proteins by immunostaining using either a Myc- or a HA-specific antibody. INX-18::HA displayed punctate localization at muscle intercellular junctions (Figure **4**, *top*). Myc::INX-11 displayed punctate localization at muscle intercellular junctions and inside muscle cells (likely corresponding to dense bodies) although size and density of the puncta at intercellular junctions appeared to be distinct from those of the other innexins (Figure **4**, *top*). An image of UNC-9::GFP in body-wall muscle is also shown for the purpose of comparison.

We previously showed that UNC-9::GFP fusion protein is functional [34]. To determine whether the fusion proteins for the remaining 5 innexins are also functional, we expressed them in body-wall muscle of corresponding mutants under the control of P*myo-3* and analyzed their effects on electrical coupling. The coupling deficiencies of 4 innexin mutants (*inx-1, inx-10, inx-11* and *inx-18*) were rescued whereas that of *inx-16* was not (Figure **4**, *bottom*). The failure for INX-16::GFP to rescue muscle coupling might result from insufficient membrane targeting/localization and/or a nonfunctional fusion protein. Interestingly, worms expressing the fusion proteins tended to display larger *G*_*j*_ compared with wild type, which was different from the observations with wild-type innexins but is reminiscent of an earlier observation with UNC-9::GFP [34].

## Discussion

The results of the present study suggest that six innexins likely assemble into two populations of gap junctions in *C. elegans* body-wall muscle. This conclusion is based on comparisons of *G*_*j*_ among wild type and various single and double mutants of the innexins. The fact that overexpressing any one of the six innexins in its wild form in body-wall muscle of a corresponding mutant did not result in a significantly higher *G*_*j*_ compared with wild type worms favors the notion that gap junctions in body-wall muscle normally consist of two or more different innexins.

The identified innexins are expected to show expression in body-wall muscle and punctate subcellular localization at muscle intercellular junctions. Indeed, muscle expression was detected for four of the six innexins when GFP was used as a reporter of innexin promoter activity, and punctate subcellular localization at muscle intercellular junctions was observed for five of the six innexins when they were expressed as either GFP- or epitope-tagged proteins. However, muscle expression was not detected for two of the six innexins; the density and size of the intercellular puncta varied considerably among the innexins when they were analyzed as fusion proteins. The failure to detect muscle expression for an innexin could be due to the absence of a regulatory element in the promoter::GFP transcriptional fusion, such as introns [40] and distal regulatory elements [41,42]. It could also be due to weak GFP expression or GFP fusion to the initiation site of a splice isoform that is not normally expressed in muscle. The apparent variability in subcellular localization could be due to interference by GFP or the epitope tag, or the use of an innexin splice isoform that is not normally expressed in body-wall muscle. Innexin genes appear to have extensive alternative splicing because previously unknown splice variants were frequently identified during our processes of isolating innexin cDNAs by RT-PCR using primer pairs based on only one previously reported isoform for each innexin (examples described in Methods). It is very likely that some splice variants were missed, especially those with alternative 5’- and 3’-ends. Therefore, the negative expression data and variable subcellular localization patterns do not challenge our main conclusion. Convincing expression and subcellular localization results may come from immunostaining with antibodies against a common region of all splice isoforms for each innexin. However, such an antibody is currently available only for UNC-9 [34].

**Figure 5:**
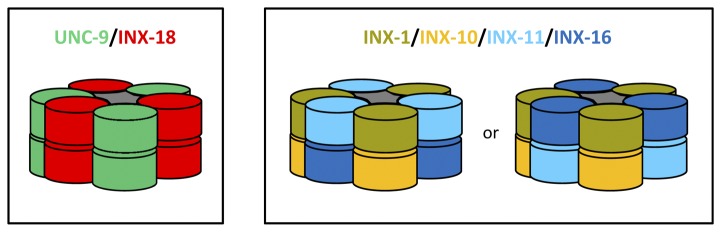
Speculative models of muscle gap junctions. These highly speculative models are used to illustrate three possible stoichiometries out of the myriad possibilities. See the text for reasons for speculating these models.

Two or more gap junction proteins could coassemble into homomeric/heterotypic, heteromeric/heterotypic, or heteromeric/homotypic gap junctions [21]. It is impossible to predict exact stoichiometries of the muscle gap junctions because the number of possible composition stoichiometries for a gap junction formed by only two innexins is as many as 196 [21]. Nevertheless, we have ventured to put together a few models based on several assumptions (Figure **5**). First, each muscle cell expresses all six innexins. Second, a heteromeric hemichannel of two different innexins is more stable in structure than that of three or four different innexins because the two different innexins may each contribute three subunits and arrange in an alternate order to form a 3-fold symmetric structure. Third, two innexins that have similar effects on *G*_*j*_ when mutated likely exist in separate hemichannels of a gap junction but are docked to each other; and a mutation of either of them would prevent the other from contributing to functional gap junctions. These models are postulated not to imply that they represent the actual stoichiometries but to show that the six innexins could be accommodated as components of two populations of gap junctions. The models have obvious limitations or caveats. For example, a few rather than all of the six innexins might be expressed in each muscle cell. There might be other innexins contributing to muscle electrical coupling because mutants of three innexins were not analyzed. Electrical coupling between other muscle cell pairs might be mediated by gap junctions of different molecular compositions because the electrophysiological analyses were restricted to selected ventral muscle cells between the neck and vulval region. Further studies may help define how various innexins assemble stoichiometrically into gap junctions in muscle.

The identification of six innexins functioning in a single cell type was unexpected and unprecedented, although such a possibility was suggested by our earlier innexin expression map [37]. It is hard to imagine that this is a phenomenon unique to *C. elegans* body-wall muscle. The findings of this study suggest that molecular compositions and stoichiometries of gap junctions in a specific cell type could be highly complex. Identification of all gap junction proteins expressed in a particular cell type and determination of the molecular stoichiometries of the gap junctions should be an essential step for fully understanding how gap junctions contribute to various physiological functions.

## Methods

### Growth and Culture of *C. elegans*

*C. elegans* hermaphrodites were grown on nematode growth medium agar plates with a layer of OP50 *Escherichia coli* inside an environmental chamber (21 °C).

### Electrophysiology

An adult hermaphrodite was immobilized on a glass coverslip by applying Vetbond^TM^ Tissue Adhesive (3M Company, MN, USA). Application of the adhesive was generally restricted to the dorsal middle portion of the animal, allowing the head and tail to sway during the experiment. A longitudinal incision was made in the dorsolateral region. After clearing the viscera, the cuticle flap was folded back and glued to the coverslip, exposing the ventral nerve cord and the two adjacent muscle quadrants. A Nikon FN1 upright microscope equipped with a 40X water-immersion objective and 15X eyepieces was used for viewing the preparation. Borosilicate glass pipettes with a tip resistance of 3~5 MΩ were used as electrodes for voltage-clamp recordings in the classical whole-cell patch clamp configuration with a Multiclamp 700B amplifier (Molecular Devices, Sunnyvale, CA) and Clampex software (version 10, Molecular Devices). Series resistance was compensated to 70-80%. To record *I*_*j*_, the membrane potential (*V*_*m*_) of both cells was held at -30 mV, from which a series of voltage steps (-110 mV to +50 mV at 10 mV intervals, 250 ms duration) were applied to one cell (Cell 1) whereas the other cell (Cell 2) was held constant to record *I*_*j*_. The voltage steps were repeated four times and automatically averaged for subsequent analyses. *I*_*j*_ was sampled at a rate of 10 kHz after filtering at 2 kHz. Junctional voltage (*V*_*j*_) was defined as *V*_*m*_ of Cell 2 minus *V*_*m*_ of Cell 1.

The extracellular solution contained (in mM) NaCl 140, KCl 5, CaCl_2_ 5, MgCl_2_ 5, dextrose 11, and HEPES 5 (pH 7.2). The pipette solution contained (in mM) KCl 120, KOH 20, Tris 5, CaCl_2_ 0.25, MgCl_2_ 4, sucrose 36, EGTA 5, and Na _2_ATP 4 (pH 7.2).

### cDNA cloning

cDNAs of the innexins were cloned through RT-PCR using primer pairs complementary to the 5’- and 3’-ends of one isoform (the longest one if more than one had been reported) for each innexin listed at the Wormbase website (www.wormbase.org. Accessed 2013 July 1). The specific cDNA isoforms used to guide the primer design were C16E9.4a for *inx-1*, T18H9.5a for *inx-10*, W04D2.3b for *inx-11*, R12E2.5 for *inx-16*, and C18H7.2a for *inx-18*. A cDNA of *unc-9* was cloned in an earlier study [33]. Two isoforms of cDNA were cloned for *inx-1*, including C16E9.4a and a novel isoform (GenBank KF137642). Three isoforms of cDNA were cloned for *inx-10*, including T18H9.5a and two novel isoforms (GenBank KF137643 and KF137644). Two novel isoforms of cDNA were cloned for *inx-11* (GenBank KF137645 and KF137646). The cloning of KF137646 was aided by using primers complementary to a novel exon identified through comparing genomic DNA sequences between *C. elegans* and *C. briggsae*, as we did for other genes [43]. The cDNAs cloned for *inx-16* and *inx-18* are identical to R12E2.5 and C18H7.2a, respectively.

### Mutant Rescue

Muscle-specific rescue was performed by injecting a plasmid containing P*myo-3* fused to the initiation site of a wild-type innexin cDNA into a corresponding innexin mutant using a standard technique [44]. A P*myo-3*::GFP plasmid (pPD118.20) was coinjected to serve as a transformation marker, which caused body-wall muscle cells to display bright green fluorescence. The specific cDNA isoforms used in these experiments were C16E9.4a for *inx-1* (plasmid wp1217), T18H9.5a for *inx-10* (plasmid p1219), W04D2.3c for *inx-11* (plasmid wp1221), R12E2.5 for *inx-16* (plasmid wp1222) and C18H7.2a for *inx-18* (plasmid p724). The muscle-specific rescue strain for *unc-9* was from an earlier study [33]. The plasmids used for testing the ability of fusion proteins to rescue muscle coupling was wp1227 for INX-1::GFP, wp1224 for INX-10::GFP, wp441 for Myc::INX-11, wp1235 for INX-16::GFP, and wp728 for INX-18::HA. *I*_*j*_ was recorded from pairs of adjacent cells showing GFP fluorescence.

### Analyses of Expression Pattern

Procedures for assessing innexin expression patterns using the homologous recombination approach are described previously [37]. To create the new promoter::GFP transcriptional fusion plasmids for *inx-1* (wp1264) and *inx-16* (wp1266), a promoter sequence before the translation initiation site of either gene was amplified by PCR with genomic DNA as a template, and used to replace P*myo-3* in pPD118.20 by cutting with Pst1 and BamH1 restriction enzymes. The sequences of the PCR primers were TGTCTCTGCTTTGCACGATT (sense) and attggatccGGCGGACAAGAACTGCAAT (antisense) for P*inx-1*, and atactgcagATGCTGGCTAGTTATATCCC (sense) and ataggatccTTTGGTGTACCCTGAAATTAA (antisense) for P*inx-16*. The sense primer for P*inx-1* did not contain a Pst1 restriction site because an internal Pst1 site in the promoter was used for the cloning. The promoter::GFP transcriptional fusions were injected individually into *lin-15*(*n765*) [45]. A *lin-15* rescue plasmid was coinjected to serve as a transformation marker. GFP fluorescence signal was imaged using a Nikon inverted microscope (TE2000-U) with a 40X objective, a FITC filter (41001, Chroma Technology Corp., Bellows Falls, VT, USA) and a Peltier cooled digital camera (F-view II; Olympus).

### Analyses of Subcellular Localization

GFP- or epitope (Myc or HA)-tagged full-length innexins were expressed in body-wall muscle under the control of P*myo-3*. The specific cDNA isoforms used in these experiments were C16E9.4a and a novel cDNA (GenBank KF137642) for INX-1::GFP (plasmids wp1218 and wp1227), T18H9.5a for INX-10::GFP (plasmid wp1224), R12E2.5 for INX-16::GFP (plasmid wp1235) and C18H7.2a for INX-18::HA (plasmid wp728). Myc::INX-11 was made by fusing a Myc sequence to the initiation site of W04D2.3a in genomic DNA (plasmid wp441). The P*myo-3*::UNC-9::GFP strain was from an earlier study [33]. INX-11 was tagged on the amino terminal whereas the remaining innexins were tagged at the carboxyl terminal. These plasmids were injected separately into *lin-15*(*n765*) [45]. A *lin-15* rescue plasmid was coinjected to serve as a transformation marker. Transgenic worms expressing a GFP-tagged innexin were imaged after immobilization whereas those expressing a Myc- or HA-tagged innexin were imaged after permeabilization with a Bouin’s fixative [46] and immunostaining first with a mouse anti-HA or a rabbit anti-Myc antibody (Invitrogen Corporation, Carlsbad, CA) and then with an Alexa Fluor® 488-conjugated goat anti-mouse or an Alexa Fluor® 594-conjugated goat anti-rabbit secondary antibody (Invitrogen Corporation). The fluorescence signals were imaged using the Nikon TE2000-U inverted microscope with a 40X or 60X objective, FITC (41001, Chroma Technology Corp.) and Texas Red (41004, Chroma Technology Corp.) filters, and the F-view II camera.

### Statistical Analyses

The apparent mean steady-state current during the last 100 ms of each voltage step was determined with Clampfit (Molecular Devices) and used to plot *I*_*j*_ *- V*_*j*_ relationship. *G*_*j*_ was determined from the slope of the *I*_*j*_ -V_*j*_ relationship at the linear portion (*V*_j_ = −30 to +30 mV) and compared among the different groups by one-way analysis of variance (*ANOVA*) followed by Bonferroni post hoc tests for pair-wise comparisons. *p* < 0.05 is considered to be statistically significant. All values are shown as mean ± se. Sample traces of *I*_*j*_ in figures are displayed at 20-mV intervals of *V*_*j*_ steps after filtering at 300 Hz for clarity. Statistical analyses and data graphing were performed using Origin (version 9.0, OriginLab Corporation, Northampton, MA).
